# Effects of Different Carbon and Nitrogen Ratios on Yield, Nutritional Value, and Amino Acid Contents of *Flammulina velutipes*

**DOI:** 10.3390/life14050598

**Published:** 2024-05-08

**Authors:** Jiandong Han, Ruixiang Sun, Chunyan Huang, Hongyan Xie, Xia Gao, Qiang Yao, Peng Yang, Jin Li, Zhiyuan Gong

**Affiliations:** 1 State Key Laboratory of Nutrient Use and Management/Key Laboratory of Wastes Matrix Utilization, Ministry of Agriculture and Rural Affairs, Institute of Agricultural Resources and Environment, Shandong Academy of Agricultural Sciences, Jinan 250100, China; hanjiandong@saas.ac.cn (J.H.); sunruixiang@saas.ac.cn (R.S.); huangchunyan@saas.ac.cn (C.H.); xiehongyan@saas.ac.cn (H.X.); yaoqiang@saas.ac.cn (Q.Y.); yangpeng@sdsnykxy.wecom.work (P.Y.); lijin@saas.ac.cn (J.L.); 2 Shandong Agricultural Technology Extending Center, Jinan 250100, China; chuchugao@163.com

**Keywords:** *Flammulina velutipes*, agronomic traits, amino acid composition, metabolomics alterations

## Abstract

The carbon-to-nitrogen (C/N) ratio in the cultivation medium significantly influences the growth rate, vigor of mycelium, yield of fruiting bodies, and their nutritional composition. Recently, agricultural and forestry wastes have been increasingly used in cultivating *Flammulina velutipes*. However, systematic research on how these materials affect the nutritional and functional properties of the fruiting bodies is lacking. This study investigated the effects of different C/N ratios on *F. velutipes* cultivation. We evaluated the agronomic traits, nutritional composition, and flavor compounds of the fruiting bodies. Our findings reveal that an optimal C/N ratio of 27:1 in the composted substrates enhances the total yield of fruiting bodies, with 25.1% soybean straw as the primary raw material. This ratio also significantly increases the levels of crude protein, total amino acids, and essential amino acids in the fruiting bodies (*p* < 0.05). Fruiting bodies from the high-nitrogen (HN) treatment showed the highest content of umami amino acids and equivalent umami concentration value. Additionally, we employed an untargeted liquid chromatography–mass spectrometry (LC-MS)-based metabolomics approach to analyze the metabolite profiles of fruiting bodies cultivated in high-nitrogen (HN), medium-nitrogen (MN), and low-nitrogen (LN) substrates. We found that the carbon–nitrogen ratio can affect the flavor and quality of fruiting bodies by regulating amino acid biosynthesis and metabolism and other related pathways. Our results suggest that a C/N ratio of 27:1 offers numerous benefits for the cultivation of *F. velutipes* with comprehensive analyses and has promising application prospects.

## 1. Introduction

*Flammulina velutipes* (Curt. ex Fr.) Sing, also known as Winter Mushroom or Enokitake, is a significant mushroom for both culinary and medicinal purposes, widely cultivated on a grand scale [1,2,3,4]. China’s edible mushroom industry has experienced significant growth, with the production of edible mushrooms increasing from 7.8 million tons in 2001 to 42.254 million tons in 2022, marking a 5.5-fold increase, as reported by the China Edible Mushroom Association. Specifically, the production of *F. velutipes* reached 2.0254 million tons in 2022. This rapid expansion has led to a significant consumption of raw materials for cultivation, resulting in a continuous increase in the cost of traditional materials and squeezing profit margins for businesses. Cottonseed hulls, corncobs, sawdust, and bagasse are the standard substrates for cultivating *F. velutipes*. However, the urgent need to explore alternative agricultural wastes as new cultivation substrates is becoming more apparent. 

Numerous studies have explored the cultivation of *F. velutipes* using various substrates [5,6], demonstrating that both yield and quality of mushrooms significantly depend on the cultivation medium [7,8,9]. The C/N ratio is a critical factor affecting mycelial growth rate, vigor, fruiting body yield, and nutritional content. Different mushroom species require specific C/N ratios for optimal mycelial growth and maximum yield [10,11]. Research using cottonseed hulls and bran found that a 30% bran addition maximized mycelial vigor, growth rate, and fruiting body biological efficiency at 123.50% [12]. Another study, utilizing sawdust, cottonseed hulls, and rice bran, achieved the highest yield of *F. velutipes* at a C/N ratio of 30:1 [13]. Investigations into varying C/N ratios, using cottonseed hulls and cotton stalks, revealed a positive correlation between the C/N ratio (ranging from 25:1 to 40:1), yield of fruiting bodies, and essential amino acid content, with the best results at a 40:1 ratio [14]. Additionally, a study employing cottonseed hulls, broadleaf tree sawdust, and bran determined that a C/N ratio of 38:1 resulted in the fastest mycelial growth and highest biological efficiency of the fruiting bodies [15].

Soybean (*Glycine max*) is a widely cultivated oilseed crop in North America (primarily the USA), Latin America (mainly Argentina and Brazil), Asia (particularly China and India), and parts of Europe [16]. According to the Ministry of Agriculture and Rural Affairs of the People’s Republic of China, China’s soybean production reached 20.28 million tons in 2022, According to Lopes et al. [17] and Martelli-Tosi et al. [18], the mass ratio productivity of straw to soybeans ranges from 1.2 to 1.5, which implies the production of about 24.34 million tons–30.42 million tons of straw in 2022. Soybean straw, a rich and renewable agricultural waste resource, offers wide availability, abundant quantity, low cost, and a short regeneration cycle [19]. It contains high levels of crude protein, lignin, cellulose, and hemicellulose. With its high C/N ratio, soybean straw serves as an important organic energy source, enriched with minerals like potassium (K) and crude protein (CP), making it highly valuable for use. At present, soybean straw is mainly utilized as feed, fuel, etc., with low effective recycling and utilization rates, resulting in a waste of resources. The purpose of the research was to clarify the effects of different C/N ratios on the yield, biological efficiency (BE), agronomic traits, and nutritional quality of *F. velutipes*, and to screen for suitable C/N ratios when cultivating *F. velutipes* from soybean straw. The research has a hypothesis that relates to the following aspects. As nitrogen content increases, the culture material is nutrient-sufficient, the growth rate of fruiting bodies becomes faster, the growth cycle is shortened, the nutritional quality is improved, and the flavor substances are enhanced. How the C/N ratio would affect the growth and development and nutrient composition of the fruiting bodies is a question that deserves to be investigated. The findings will support the use of soybean straw in *F. velutipes* cultivation and contribute to improving the yield and quality of the fruiting bodies.

## 2. Materials and Methods

### 2.1. Inoculum Source and Spawn Preparation

The *F. velutipes* strain for this study was obtained from Shandong Youshuo Biotechnology Co., Ltd., located in Jining, China. The stock culture was maintained on PDA (Potato Dextrose Agar) medium, while the spawn preparation involved a mix of 18% wheat bran, 80% sawdust, and 2% lime. This substrate was incubated for 30 days at 25 °C in darkness and subsequently stored at 4 °C for later use.

### 2.2. Composting Process and Mushroom Cultivation

The soybean straw was added in the same proportion in each formulation, and the carbon-to-nitrogen ratio was adjusted by adjusting the addition proportion of corn cobs, rice bran, cottonseed husk, and brewer’s grains. Among them, rice bran and brewing grains were the main sources of nitrogen, with nitrogen contents of 1.73% and 4.00%, respectively. According to the cultivation medium formulation (Table 1), C/N ratios of 22:1, 27:1, and 37:1 were designated as high-nitrogen (HN), medium-nitrogen (MN), and low-nitrogen (LN) treatments, respectively, and were used to cultivate *F. velutipes* using a commercial production model [20]. The medium was bottled, five holes were punctured for ventilation, and then the medium was sterilized at 121 °C for 2 h. Each bottle received 30 mL of liquid spawn and was placed in a dark incubation chamber at 18 °C with controlled carbon dioxide levels. Upon full colonization by the fungus, the surface was scraped (0.3–0.5 cm) and then maintained at 14 °C and 90–92% relative humidity. Lighting commenced on the third day. After primordia appeared, humidity was slightly reduced. When fruiting bodies grew 2–3 cm beyond the bottle’s mouth, a paper collar was added, and the temperature was kept at 8–12 °C until harvest. Three replicates were conducted for each treatment, with each replicate comprising 12 bottles.

For sampling, mushroom stalks shorter than 3 cm were classified as young mushrooms (YG), and those longer than 10 cm as mature mushrooms (CG). Six bottles showing uniform growth per treatment were selected for harvest. The fruiting bodies were removed, the substrate detached, and then the fruiting bodies were placed in 2.5 mL RNAase-free centrifuge tubes, quickly frozen in liquid nitrogen, and stored at −80 °C. Additionally, another six bottles per treatment showing uniform growth were chosen for agronomic trait analysis. These fruiting bodies were dried in a 56 °C oven, labeled post-drying, and stored in sealed bags for nutrient analysis.

### 2.3. Agronomic Traits

Fresh fruiting bodies from each treatment were collected for analysis. Yield (g/bottle) was determined as the fresh weight of mushrooms harvested from each bottle during the first flush. Biological efficiency (BE) was calculated as the percentage of the fresh weight of mushrooms per bottle divided by the dry weight of the corresponding substrate, multiplied by 100%. Additionally, agronomic characteristics of individual fruits were assessed, including fruiting body count, cap thickness, cap diameter, stipe length, and stipe diameter.

### 2.4. Main Nutritional Qualities

Dried fruiting bodies from each treatment were pulverized, passed through a 90-mesh sieve, and stored at 4 °C for subsequent analysis. The contents of crude protein, crude fiber, crude fat, and crude polysaccharides were measured. The assay was performed with reference to the methods of Filipa S [21], Lu et al. [22]. An automatic Kjeldahl nitrogen analyzer (Kjeltec8400, FOSS, Hilleroed, Denmark) was used to determine crude protein content. Crude fiber content was assessed using the acid–base digestion method. Crude fat content was measured through Soxhlet extraction. Finally, crude polysaccharide content was determined by ethanol precipitation.

### 2.5. Assay of Amino Acid Composition

The amino acid composition of dried fruiting bodies was analyzed using an automatic amino acid analyzer (L-8800, Hitachi, Tokyo, Japan) [7]. Briefly, 0.03 g of mushroom powder was hydrolyzed in a threaded test tube with 10 mL of 6 mol/L HCl and 5 mg/mL phenol at 110 °C for 22 h. The hydrolysate was then filtered into a 50 mL volumetric flask and diluted with deionized water. A total of 2 mL of the hydrolysate was dried at 40 °C, redissolved in 2 mL deionized water, and this drying and dissolving process was repeated three times. Finally, the dried samples were dissolved in 2 mL of 0.02 mol/L HCl and filtered through a 0.22 μm filter membrane for analysis.

### 2.6. Extraction of Metabolites from Samples

Tissues (100 mg) were individually ground with liquid nitrogen and the homogenate was resuspended with prechilled 80% methanol by well vortex. The samples were incubated on ice for 5 min and then were centrifuged at 15,000× *g*, 4 °C for 20 min. Some of the supernatant was diluted to a final concentration containing 53% methanol by LC-MS-grade water. The samples were subsequently transferred to a fresh Eppendorf tube and then were centrifuged at 15,000× *g*, 4 °C for 20 min [23]. A 20 µL volume from each prepared sample was utilized for quality control (QC) and quality assurance (QA) in order to rectify deviations and errors caused by the analytical instrument.

Liquid chromatography analysis was performed using a Vanquish Ultra-High-Pressure Liquid Chromatography (UHPLC) system (Thermo Fisher Scientific, Waltham, MA, USA) with an ACQUITY HSS T3 UPLC column (150 × 2.1 mm, 1.8 microns) (Waters, Milford, MA, USA). The column temperature was set at 40 °C, with a flow rate of 0.25 mL/min and a sample injection volume of 2 µL. The elution gradient employed 0.1% formic acid in acetonitrile (C) and 0.1% formic acid in water (D). Compounds were identified using liquid chromatography–mass spectrometry (LC-ESI (+)-MS). A 2% carbohydrate sample was introduced from 0 to 1 min, with C increasing from 2% to 50% from 1 to 9 min, reaching 50% to 98% between 9 and 12 min, and holding at 98% from 12 to 13.5 min. From 13.5 to 14 min, C returned to 2%, and from 14 to 20 min, it remained at 2%. For LC-ESI (−)-MS, the gradient components were acetonitrile (A) and 5 mM ammonium formate (B), with A varying similarly to C in the positive mode.

Electrospray ionization tandem mass spectrometry was performed on an Orbitrap Exploris 120 (Thermo Fisher Scientific) to detect metabolite mass spectra. Parameters included a cover pressure of 30 mbar, auxiliary gas flow of 10 bar, ESI (+) discharge potential of 3.50 kV, ESI (−) discharge potential of 2.50 kV at 325 °C, MS1 scan range of 100–1000, resolution of MS1 at 60,000 FWHM, loop data correlation of 4, mass spectrometry resolution of 15,000 FWHM, standard collision energy of 30%, and an automatic dynamic clearance time.

### 2.7. Statistical Analysis

All data are presented as mean ± standard deviation (mean ± SD). One-way ANOVA, conducted using SPSS (v.25.0), tested the significance of different treatments. The Student’s *t*-test analyzed significant differences in average values for the same flush across treatments, with *p* < 0.05 indicating statistical significance.

To utilize features and align retention times, raw data were converted to mzXML format using MS Convert from the ProteoWizard software suite (v3.0.8789). MS/MS data were matched with databases such as HMDB, MassBank, LIPID MAPS, mzCloud, and the Kyoto Encyclopedia of Genes and Genomes (KEGG) to accurately identify metabolites with a mass accuracy of <30 ppm. Data normalization, using robust LOESS signal correction (0C-RLSC), compensated for systematic bias. Only ion peaks with a relative standard deviation (RSD) of less than 30% in the QC were retained after normalization to ensure accurate metabolite identification.

The R software package RoPLS conducted principal component analysis (PCA) and orthogonal projections to latent structures discriminant analysis (OPLS-DA). PCA identified patterns of intra-group clustering and inter-group segregation, while OPLS-DA further explored inter-group distinctions. Data were scaled, and score plots, load plots, and sPlots illustrated differences in metabolite composition between samples. A permutation test for overfitting verified the method’s suitability. OPLS-DA identified differential metabolites using variable importance in projection (VIP) scores and *p*-values from the combination of OPLS-DA and fold change (FC), highlighting key factors affecting classification results. Significant differences in metabolite content were indicated by *p*-values < 0.05 and VIP values > 1. MetaboAnalyst 4.0 software analyzed metabolite pathways, integrating pathway topology with analysis methods. KEGG pathway analysis revealed the physiological functions of pathways at a higher level, with metabolites and pathways visualized using KEGG drawing software.

## 3. Results and Discussion

### 3.1. Effects of C/N Ratios on Agronomic Traits of Mushrooms

The growth and development of *F. velutipes* were influenced by different C/N ratios in the culture medium (Table 2). With varying C/N ratios, the composition and quantity of the culture medium adjusted, notably decreasing the volume of low-nitrogen media in the bottle. The medium-nitrogen (MN) treatment group had the shortest growth cycle of 42.26 days and concentrated harvesting period of *F. velutipes*. This group also had the highest yield, at 356.10 ± 16.18 g/bottle, significantly surpassing other groups (*p* < 0.05). The biological efficiency of the medium-nitrogen group was comparable to the low-nitrogen group (*p* > 0.05) but significantly exceeded the high-nitrogen group (*p* < 0.05).

Different C/N ratios also significantly affected agronomic traits (Table 3). The medium-nitrogen group exhibited the longest stipe length at 158.74 ± 5.01 mm, noticeably longer than other groups (*p* < 0.05), while pileus thickness showed no significant difference across groups (2.77 to 2.86 mm) (*p* > 0.05). The optimal harvesting period is when the stem length reaches 150–170 mm; it shows that the commodity properties of medium-nitrogen treatment are better. The diameter of the stipe and pileus in the medium-nitrogen group did not exhibit a significant difference compared to that of the low-nitrogen group, but was significantly higher than that of the high nitrogen group. There was no significant disparity in the number of fruiting bodies between the high- and medium-nitrogen groups; however, both were notably higher than that of the low-nitrogen group. The optimal C/N ratio for *F. velutipes*, found to be 30/1 [6], was most closely achieved with the medium-nitrogen treatment, reaching a maximum biological efficiency of 108%. A moderately lower C/N ratio benefits mycelial growth in oyster mushrooms [9]. Usually, formulations with a high carbon content in the substrate contain fewer nitrogenous materials and the culture material is more air-permeable, which favors mycelial growth, in addition to a possible correlation with the nutrient content of the raw material. The physicochemical properties of substrates play a crucial role in mushroom yield and nutritional quality [24]. The water content, particle size, and capacity of different compositions of culture materials will affect the growth rate of mycelium and the utilization efficiency of mycelium on substrate; the nutrient compositions of different kinds of culture materials differ greatly, such as protein, organic matter, etc., so there is a difference in the absorption, utilization, and transformation of nutrients by mycelium, which affects the growth of the substrate and the nutrient composition, and thus affects the yield and quality of the fruiting body. Thus, adjusting the C/N ratio can enhance both yield and quality of the fruiting body, aligning with findings from other mushroom cultivation studies [25].

### 3.2. Nutritional Quality of Fruiting Bodies

Mushrooms are rich in nutrients, and their nutritional profiles vary depending on the cultivation substrates used [26]. The ratios of carbon to nitrogen play a crucial role in determining the levels of crude protein and fat in the fruiting bodies, with significant increases observed as the nitrogen content rises (*p* < 0.05). Specifically, the crude protein content escalates from 15.52% to 22.09% and the fat content from 1.02% to 1.37% as nitrogen levels increase (*p* < 0.05) (Table 4)**.** Studies have shown that substrates enriched with nitrogen enhance protein concentrations in mushrooms of the *Pleurotus* genus [27]. The addition of nitrogen source in *F. velutipes* significantly increased the total protein content, and the total protein content was negatively correlated with the C/N ratio [28].

Additionally, the crude fat content is significantly influenced by the mushrooms’ characteristics and the growth conditions, particularly the nitrogen sources in the substrate [29]. The highest-nitrogen group exhibited the most substantial protein and fat contents, at 22.09% and 1.37%, respectively, significantly surpassing other groups (*p* < 0.05), corroborating prior research.

The total sugar content peaked in the medium-nitrogen group at 31.68%, slightly ahead of the low-nitrogen group, with no notable difference between them. Yet, both significantly exceeded the high-nitrogen group’s sugar content (*p* < 0.05). Conversely, the polysaccharide content was lowest in the medium-nitrogen group, differing significantly from the rest (*p* < 0.05). These findings suggest that a balanced addition of nitrogen can significantly enhance the nutritional quality of *F. velutipes*.

### 3.3. Amino Acid Composition

Mushrooms are known for being a rich source of protein, with an amino acid composition comparable to animal and dairy products [30]. Dietary amino acids can enhance the innate and adaptive immunity in both humans and animals [31]. Amino acids, which have various physiological functions, are crucial for sustaining life [32]. In our study, all 16 tested amino acids were present in the fruiting bodies cultivated on three different culture media. The amino acid profiles for high-nitrogen (HN), medium-nitrogen (MN), and low-nitrogen (LN) treatments are detailed in Table 5. The total amino acid (TAA), essential amino acid (EAA), and non-essential amino acid (NEAA) contents were significantly affected by varying C/N ratios (*p* < 0.05). TAA content ranged from 10.45 g to 16.81 g per 100 g dry weight across treatments, with the highest value observed in the HN group (16.81 g/100 g), significantly surpassing the MN and LN groups (*p* < 0.05). The LN group’s amino acid content was 6.36 g and 5.91 g lower per 100 g dry weight than that of the HN and MN groups, respectively. EAA content varied from 4.13 to 7.65 g/100 g dry weight, with the MN group recording the highest value (7.65 g/100 g), significantly exceeding the other groups (*p* < 0.05).

*F. velutipes*, also known as the benefiting intelligence mushroom [33], is a rich source of lysine and arginine, both of which are beneficial for children’s growth and cognitive development. Except for alanine, methionine, tyrosine, and proline, the concentration of the other 13 amino acids increased with nitrogen concentration. The lysine content in the high-nitrogen treatment group showed an increase of 6.87% and 53.31% compared to the medium-nitrogen and low-nitrogen treatment groups, respectively, while the arginine content in the high-nitrogen treatment group exhibited an increase of 28.28% and 107.88% compared to that of the medium-nitrogen and low-nitrogen treatment groups, respectively. A proper C/N ratio can enhance the content of specific amino acids in the substrate, and a suitable C/N ratio can be chosen based on yield or quality requirements. The study also noted that mushroom fruiting bodies cultivated in tea residue-enriched medium, which is more nutritious, showed increased total and essential amino acid contents as the proportion of tea residue in the substrate increased [34]. Among all treatments, the HN group had the highest TAA and NEAA contents (16.81 g/100 g and 9.51 g/100 g, respectively). In summary, appropriate C/N ratio can enhance the crude protein content, amino acid composition, and flavor of *F. velutipes* fruiting bodies.

Amino acids are major contributors to mushroom flavor [35], classified into umami, sweet, bitter, and tasteless categories based on their flavor profiles. Umami and sweet amino acids are key flavor components [9,36]. Glutamic acid (Glu) and aspartic acid (Asp), akin to monosodium glutamate, are responsible for mushrooms’ umami taste [9,37]. This study found that high nitrogen significantly improved the Glu and Asp contents of the fruiting bodies (3.161 g/100 g and 1.176 g/100 g, respectively), enhancing their taste. The HN treatment yielded the highest levels of umami and sweet amino acids (4.337 g/100 g and 3.700 g/100 g, respectively), suggesting mushrooms grown with high nitrogen levels are more flavorful. In different treatments, the amino acids of the umami taste in the fruiting bodies of *F. velutipes* were higher than the amino acids of the sweet taste, which was consistent with the results of Beluhan and Ranogajec [10]. The arginine content in the fruiting bodies of *F. velutipes* in the high-nitrogen group exceeded previously reported levels (0.49–3.88 mg/g) [38,39,40].

In the process of adjusting different C/N ratios, raw materials with higher nitrogen content were mostly used to adjust C/N ratios, and carbohydrates and trace elements in the culture materials changed when the ratio of the culture materials changed, and the effect on the yield and quality of the fruiting bodies was the result of the joint action of many factors, among which the carbon and nitrogen ratio played a major role; in future experiments, we can try to add simple carbon or nitrogen sources to adjust C/N ratios in order to study the effect of different carbon and nitrogen ratios on the growth, development, and quality of the fruiting bodies.

### 3.4. Changes in Different C/N Ratios Lead to Metabolomics Alterations

**Figure 1:**
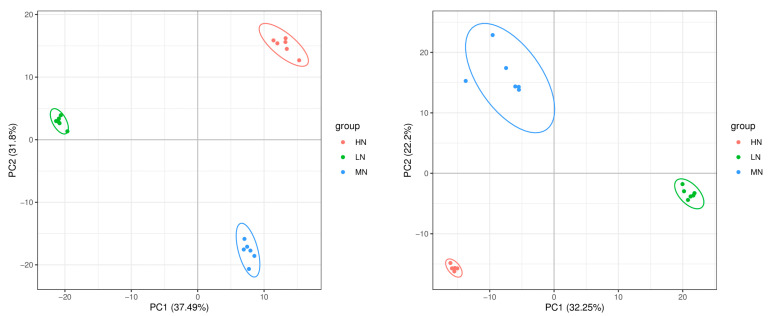
Plots of metabolite PCA results in positive and negative ion mode (**left**: negative ion mode; **right**: positive ion mode).

An untargeted liquid chromatography–mass spectrometry (LC-MS)-based metabolomics approach characterized metabolite profiles from high-nitrogen (HN), medium-nitrogen (MN), and low-nitrogen (LN) substrate fruiting bodies of *F. velutipes*. Principal component analysis confirmed the repeatability among replicates (Figure 1). Metabolites showing significant differences were identified using variable importance in projection (VIP) values > 1.0 and *p* < 0.05 (two-tailed Student’s *t*-test).

**Figure 2:**
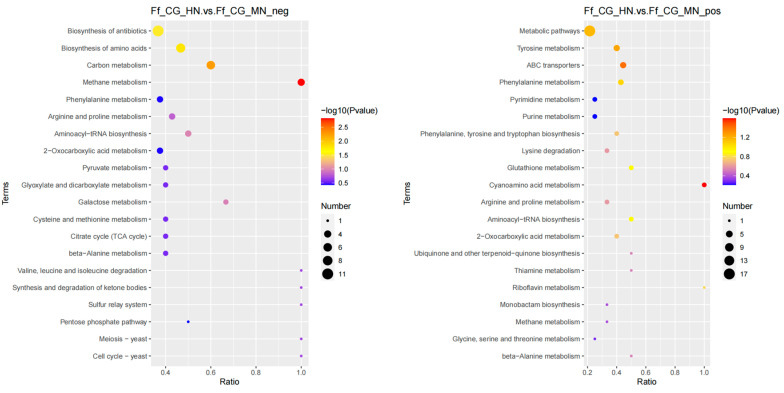
KEGG enrichment analysis of metabolites in mature fruiting bodies treated with high and medium nitrogen. (**left**: negative ion mode; **right**: positive ion mode).

**Figure 3:**
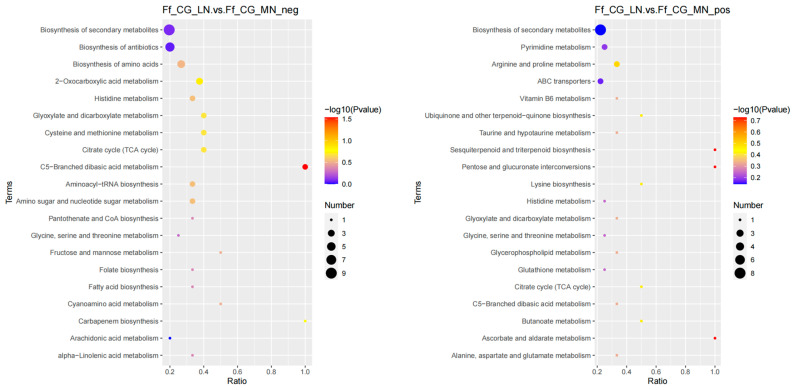
KEGG enrichment analysis of anion and cation metabolites in mature fruiting bodies treated with low and medium nitrogen. (**left**: negative ion mode; **right**: positive ion mode).

**Figure 4:**
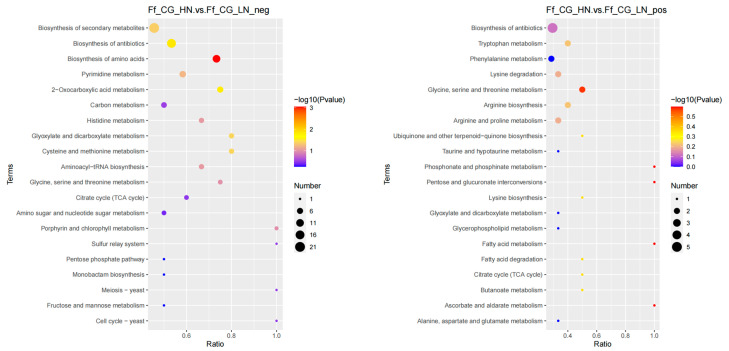
KEGG enrichment analysis of anion and cation metabolites in mature fruiting bodies treated with high and low nitrogen. (**left**: negative ion mode; **right**: positive ion mode).

In the differential analysis between HN and MN, 188 metabolites were identified in positive ion mode, enriching 24 metabolic pathways, while 161 metabolites in negative ion mode enriched 39 pathways; the differential metabolites are predominantly enriched in pathways related to secondary metabolite biosynthesis and amino acid biosynthesis (Figure 2). The comparison between HN and LN revealed 229 metabolites in positive ion mode across 22 pathways, and 167 metabolites in negative ion mode across 31 pathways. Differential metabolites were mainly enriched in amino acid biosynthesis, secondary metabolite biosynthesis pathway, followed by glycine, serine, and threonine metabolism, tryptophan metabolism, arginine biosynthesis, pyrimidine metabolism pathway (Figure 3). For LN versus MN, 222 metabolites were identified in positive ion mode enriching 22 pathways, and 159 metabolites in negative ion mode enriching 21 pathways. The differential metabolites were predominantly enriched in the secondary metabolite biosynthesis pathway, followed by amino acid biosynthesis, arginine and proline metabolism, purine metabolism, ABC transport protein pathways, and other related pathways (Figure 4).

For detailed analysis, differential metabolites with |log2 FC| > 2 were selected. In positive ion mode, metabolites such as L-(+)-Citrulline and 3,4-Dihydroxy-4-(4-methoxyphenyl)-1,2,3,4-Tetrahydroquinolin-2-one were examined. The study noted a decrease in the expression of these substances as nitrogen content increased. Tetrahydrocannabinol (THC), significantly expressed under low-nitrogen conditions, is known for its use in treating nausea and stimulating appetite but is also a controlled psychoactive compound [41]. N-Acetylornithine, crucial for arginine biosynthesis, showed decreased expression with increased nitrogen, affecting fruiting body quality [42]. Significant differences were also observed in the expression of alpha-Ketoglutaric acid in the arginine biosynthesis pathway, particularly between HN and LN, where it was downregulated by 1.67 times. Alpha-Ketoglutaric acid, an essential intermediate in the tricarboxylic acid cycle, plays a crucial role in nitrogen metabolism and organism growth [43].

Increased nitrogen levels were found to contribute to the accumulation of some substances, such as Tanespimycin, an Hsp90 inhibitor that affects tumor cell proliferation [44]. Tanespimycin expression varied significantly across different nitrogen levels, illustrating the impact of appropriate nitrogen content on its expression. Similarly, DL-Citrulline expression was notably different across treatments, being upregulated in HN versus LN and HN versus MN comparisons. As nitrogen content increased, DL-Citrulline expression was inhibited, highlighting its role in the urea cycle when combined with aspartic acid [45].

Varied C/N ratios in the culture medium significantly influenced the metabolite contents in *F. velutipes* fruiting bodies. These distinct metabolites primarily pertained to amino acid biosynthesis and metabolism and were found to be enriched in pathways associated with secondary metabolite biosynthesis, purine and pyrimidine metabolism. Consequently, these factors impacted the amino acid and other metabolite levels within the substrates, ultimately influencing their flavor and overall quality.

*F. velutipes* has edible and medicinal value; with the development of society, people’s demand for the product will be diversified, and the C/N ratio and other factors in the formula can be adjusted according to the market demand, in order to enhance the flavor, nutrition, and active substance content of the fruiting bodies. C/N ratios had significant effects on the changes in metabolites in fruiting bodies, and the differential metabolite species exceeded hundreds and were enriched in different pathways, related to amino acid synthesis and metabolism, energy metabolism, and other pathways, which led to the changes in amino acid content in fruiting bodies and the growth rate of the fruiting bodies, providing a reference for the study of the effects of C/N ratios on the growth and development of substrates.

## 4. Conclusions

The effects of different C/N ratios in the culture medium on the growth and development, nutritional quality, and metabolites of *F. velutipes* were investigated by adjusting the contents of corncob, rice bran, cottonseed hulls, and brewer’s dregs to regulate the carbon and nitrogen ratios in the formulation using 25.1% soybean straw as the raw material. The results showed that an appropriate C/N ratio (27:1) in the culture medium could shorten the growth cycle and improve the yield of *F. velutipes*. In terms of agronomic traits, an appropriate C/N ratio could improve stipe length and had no significant effect on stipe and pileus diameter and pileus thickness; in terms of nutritional quality, the amount of crude protein, crude fat, and total amino acids in the fruiting bodies showed a significant upward trend with increasing nitrogen content of the culture medium; except for methionine, tyrosine, phenylalanine, and proline, the content of the remaining amino acids increased with increasing nitrogen content. In terms of metabolites, different nitrogen treatments resulted in many types of differential metabolites in the fruiting bodies, and the differential metabolites were mainly enriched in amino acid biosynthesis and amino acid metabolic pathways, which led to differences in amino acid contents in the fruiting bodies of *F. velutipes.* In actual production, we can adjust the appropriate C/N ratio for the production of *F. velutipes* according to the market demand for yield and quality.

## Figures and Tables

**Table 1:** Test formulations with different C/N ratios.

Treatment	Ingredient Composition of Substrates (%)							
	Soybean Straw	Corncob	Rice Bran	Cottonseed Hull	Brewer’s Grains	CaCO_3_	Scallop Shell Powder	C/N
HN	25.1	18.84	32.2	6.18	15.74	0.87	1.07	22:1
MN	25.1	18.82	41.88	6.18	6.18	0.87	1.07	27:1
LN	25.1	40.76	32.2	0	0	0.87	1.07	37:1

HN: high-nitrogen; MN: medium-nitrogen; LN: low-nitrogen.

**Table 2:** Influence of various C/N ratios on growth and development of *F. velutipes.* Distinct lowercase letters indicate significant differences at *p* < 0.05.

Treatment	Culture Medium Wet Weight (g/bottle)	Culture Medium Dry Weight (g/bottle)	Growth Cycle (d)	Total Yield (g/bottle)	Biological Efficiency (%)
HN	890.00	329.63	44.75 ± 1.04 a	317.08 ± 14.98 b	96.19 ± 4.54 b
MN	890.00	329.63	42.26 ± 0.50 b	356.10 ± 16.18 a	108.03 ± 4.91 a
LN	830.00	307.41	44.73 ± 1.01 a	315.94 ± 15.26 b	102.78 ± 4.96 ab

HN: high-nitrogen; MN: medium-nitrogen; LN: low-nitrogen.

**Table 3:** Impact of various C/N ratios on agronomic traits of *F. velutipes.* Distinct lowercase letters indicate significant differences at *p <* 0.05.

Treatment	The Length of Stipe (mm)	The Diameter of Stipe (mm)	The Diameter of Pileus (mm)	The Thickness of Pileus (mm)	The Number of Fruiting Body (Number/Bottle)
HN	146.01 ± 8.62 b	2.60 ± 0.38 b	10.50 ± 1.46 b	2.77 ± 0.22 b	502.00 ± 26.19 ab
MN	158.74 ± 5.01 a	2.89 ± 0.39 a	11.57 ± 1.70 a	2.79 ± 0.31 b	507.67 ± 26.52 a
LN	138.96 ± 4.87 c	2.90 ± 0.32 a	10.93 ± 1.90 ab	2.86 ± 0.30 b	469.5 ± 34.38 b

HN: high-nitrogen; MN: medium-nitrogen; LN: low-nitrogen.

**Table 4:** Effects of various C/N ratios on the nutritional quality of fruiting bodies. Values in columns with differing letters indicate significant differences (*p* < 0.05).

Treatment	Crude Protein (%)	Crude Polysaccharide (%)	Total Sugar (%)	Ash (%)	Crude Fat (%)
HN	22.09 ± 0.05 a	3.97 ± 0.01 a	29.08 ± 0.38 b	5.71 ± 0.02 b	1.37 ± 0.01 a
MN	19.51 ± 0.22 b	3.40 ± 0.04 b	31.68 ± 0.43 a	6.00 ± 0.09 a	1.13 ± 0.01 b
LN	15.52 ± 0.02 c	3.94 ± 0.06 a	31.07 ± 0.29 a	5.64 ± 0.13 b	1.02 ± 0.01 c

HN: high-nitrogen; MN: medium-nitrogen; LN: low-nitrogen. Varied lowercase letters denote discrepancies across treatments within the same flush. Data are presented as mean ± SD (n = 3).

**Table 5:** Effects of different nitrogen content on amino acid content in *F. velutipes* fruiting bodies. Values within each row that are followed by different letters indicate significant differences (*p* < 0.05).

Amino Acid	Content (g/100 g Dry Weight)		
	HN	MN	LN
^1,6^Asp	1.176 ± 0.003 a	1.033 ± 0.006 b	0.824 ± 0.002 c
^2,5^Thr	0.602 ± 0.001 a	0.588 ± 0.003 b	0.549 ± 0.010 c
^2,6^Ser	0.714 ± 0.001 a	0.661 ± 0.004 b	0.594 ± 0.019 c
^1,6^Glu	3.161 ± 0.012 a	2.848 ± 0.020 b	1.999 ± 0.039 c
^2,6^Gly	0.688 ± 0.003 a	0.650 ± 0.005 b	0.504 ± 0.029 c
^2,6^Ala	0.966 ± 0.003 a	0.960 ± 0.008 a	0.681 ± 0.037 b
^3,5^Val	0.752 ± 0.003 a	0.730 ± 0.005 b	0.682 ± 0.006 c
^3,5^Met	2.599 ± 0.026 b	3.090 ± 0.028 a	0.415 ± 0.023 c
^3,5^Ile	0.616 ± 0.002 a	0.598 ± 0.005 b	0.468 ± 0.001 c
^3,5^Leu	1.097 ± 0.001 a	1.055 ± 0.008 b	0.791 ± 0.003 c
^4,6^Tyr	0.762 ± 0.002 a	0.749 ± 0.009 a	0.765 ± 0.011 a
^3,5^Phe	0.683 ± 0.001 a	0.696 ± 0.006 a	0.602 ± 0.012 b
^4,5^Lys	0.949 ± 0.004 a	0.888 ± 0.008 b	0.619 ± 0.006 c
^3,5^His	0.444 ± 0.002 a	0.404 ± 0.003 b	0.146 ± 0.005 c
^2,6^Pro	0.730 ± 0.006 a	0.733 ± 0.001 a	0.396 ± 0.023 b
^3,6^Arg	0.871 ± 0.006 a	0.679 ± 0.003 b	0.419 ± 0.003 c
Umami	4.337 ± 0.015 a	3.881 ± 0.026 b	2.823 ± 0.041 c
Sweetness	3.700 ± 0.014 a	3.592 ± 0.021 a	2.724 ± 0.118 b
EAA	7.298 ± 0.038 b	7.645 ± 0.063 a	4.126 ± 0.061 c
NEAA	9.512 ± 0.038 a	8.717 ± 0.059 b	6.328 ± 0.168 c
Total amino acid	16.810 ± 0.076 a	16.362 ± 0.122 b	10.454 ± 0.229 c

Umami: Umami Amino Acids; Sweetness: Sweet Amino Acids; Bitterness: Bitter Amino Acids; Tasteless: Tasteless Amino Acids; EAA: Essential Amino Acids; NEAA: Non-Essential Amino Acids; TAA: Total Amino Acids. Categories: 1—Umami; 2—Sweetness; 3—Bitterness; 4—Tasteless; 5—EAA; 6—NEAA. HN: high-nitrogen; MN: medium-nitrogen; LN: low-nitrogen. Significant difference means that the same amino acid is significantly different between treatments. Data are presented as mean ± SD (n = 3).

## Data Availability

The datasets used and analyzed during the current study are available from the corresponding author upon reasonable request.
